# Trauma, Terror, and Toothpaste: Exploring Memories for Dental Visits Across a Range of Patient Fear

**DOI:** 10.3390/oral5030065

**Published:** 2025-09-02

**Authors:** Kelly A. Daly, Jennie Ochshorn, Richard E. Heyman, Ronni D. Lipnitsky, Suher Baker, Adrianna O. Rozbicka, Sidhant Athilat, Allan Pike

**Affiliations:** 1 Family Translational Research Group, Center of Oral Health Policy and Management, New York University College of Dentistry, New York, NY 10010, USA; 2 Brooker Memorial Institute Pediatric Dental Center, Torrington, CT 06790, USA; 3 Dr. Pike Dentistry for Children PC, Portland, OR 97221, USA

**Keywords:** emotional fear memory, dental fear, cognitive vulnerability

## Abstract

**Background/Objectives:**

Emotional fear memories are increasingly recognized as contributors to the development of specific fears and phobias. Despite this, relatively little dental fear research has specifically focused on patient memories or their potential role in the etiology of dental fear.

**Methods:**

This two-study paper employs qualitative thematic analysis of memories for dental visits among traumatized patients (study 1) and the general patient population (ranging from endorsing no dental fear to severe fear). Recollections were evaluated based on the characteristics (i.e., sensory descriptors, affectively laden, intrusive) of emotional fear memories (studies 1 and 2) and according to a modified cognitive vulnerability model of dental fear (study 2).

**Results:**

Characteristics of emotional fear memories were ubiquitous across recollections of individuals who endorsed traumatic dental visits in childhood. Among the general patient population, these characteristics and cognitive vulnerability themes (particularly perceptions of the visit and dentist as dangerous and untrustworthy) were more prevalent in the earliest remembered visits for fearful individuals. When individuals were asked to recall their worst dental visits, emotional fear memory characteristics and vulnerability perceptions were evident across the spectrum of current fear (none to severe).

**Conclusions:**

This study contributes to nascent work examining memory in specific fears and phobias and suggests that worst recollections across a general sample share many of the characteristics that might otherwise imply vulnerability for anxiety. We recommend that dental practices universally screen patients for fear, inquire about past negative experiences, partner with patients to minimize evoking their specific vulnerabilities, and diligently implement these personalized care plans.

## 1. Introduction

Emotional fear memories (i.e., vivid, affective, sensory memories that tend to recur spontaneously) are a hallmark of post-traumatic distress and increasingly recognized as sequelae of other anxiety disorders (e.g., obsessive–compulsive disorder, social phobia) [1,2]. Developing in response to trauma or (perceived) extreme danger, emotional fear memories are believed to result from a unique memory consolidation process modulated by threat-responsive neurobiological factors at encoding [3]. Despite the robust relationship between prior traumatic dental experiences and ongoing dental anxiety [4], little research has considered the role of memory in the development and maintenance of dental fear [1]. Dental fear and anxiety (DFA), referred to henceforth as dental fear, refers broadly to a cluster of fear-induced negative symptoms arising in response to dental stimuli (i.e., dentists, procedures, tools, and settings) [5]. Dental fear includes cognitive (e.g., catastrophic thoughts about what could go wrong), emotional (e.g., physiological arousal), and behavioral (e.g., avoidance, escape) symptoms on a spectrum of severity and impairment [5].

Emotional fear memories are phenomenologically distinct among memories, even among those associated with otherwise stressful life events (e.g., an expected loss) and strong negative emotions (e.g., experiences of embarrassment, grief [6–8]). Specifically, emotional fear memories are characterized by intensity, vividness, and sensory-perceptual elements (visual, auditory, tactile, and olfactory) and are highly affectively laden (e.g., evocative of fear, helplessness, horror, disgust, rage, or pain) [6,9,10]. They are fleeting, sometimes fragmented, and may lack expected contextual details, providing a visceral glimpse of a moment in time [9,10]. Narrative research supports that the vivid sensory-perceptual and affective elements that describe emotional fear memories that recur spontaneously (i.e., intrusions) also characterize emotional fear memories that can be deliberately recalled [11].

The literature is equivocal regarding whether memory is implicated in the etiology and maintenance of dental fear [12–14]. Several large-scale dental fear reviews [15,16] fail to mention any potential role of memory, whereas others suggest repeated evocation of negative prior memories is implicit in the condition [17]. The general lack of attention to memory in dental fear research is attributable to several factors: (a) equifinality in the etiology of dental fear, as half of individuals do not have an aversive dental experience predating fear [18]; (b) the presumed importance of cognitive (memory) biases [15], rather than emotional fear memories with a clear relation to a catalyst event; and (c) the insidious nature of intrusive memories, which patients often do not think to report without direct cuing [12].

Despite this, a small body of existing research suggests that emotional fear memory plays a mechanistic role in dental fear for some patients. More than half of individuals with high dental fear have experienced a prior traumatic dental event [19], most commonly during childhood or adolescence [20]. Anxious patients who have experienced an aversive dental event exhibit elevated trauma symptoms, including endorsing recurring memories of the incident [19,21]. A large-scale study about specific phobias (the most severe, debilitating manifestation of fears) indicated that individuals with dental phobia reported more frequent and severe intrusive recollections of fear-related events than did individuals with all other common phobias (e.g., thunder, small spaces, heights, spiders) [22].

Furthermore, a few studies have focused directly on memories as related to dental fear. In two studies, fearful individuals, compared with controls, endorsed experiencing memories of dental visits that included physical sensations (e.g., heart beating, tears); sights, sounds, smells, or tastes; and emotion or mood-related content on a rating scale [21,23]. Moreover, endorsed characteristics of negative memories were significantly associated with elevated trauma symptoms within both samples [21,23]. Qualitative coding of brief descriptions for a subsample [21] indicated that negative memories most often involved recall of pain and upsetting dentist behaviors (e.g., acting impatient; scolding [21]). Across both studies, positive memories of dental visits were relatively common, but did not convey any protective effects against current dental fear or trauma symptoms [21,23]. In a large survey study, vivid sensory images of dental visits were found to be ubiquitous among dental patients, regardless of anxiety; however, the specific content of the imagery and associated distress varied as a function of participants’ dental anxiety. Follow-up interviews with a small subsample suggested that for those high in dental anxiety, imagery was more fear-provoking, evocative of uncomfortable sensations, and related to past visits; for those low in anxiety, the imagery tended to focus on reassuring and positive aspects of appointments [14]. Finally, in one additional study, individuals with dental phobia tended to report memory of a specific experience as a catalyst for their anxiety; they also rated their dental memories as more vivid, disturbing, and re-lived than did controls [1].

### 1.1. Cognitive Vulnerability Model

Despite the relative dearth of research on memory in dental fear, cognitive models of fear acquisition have received significant attention. According to the cognitive vulnerability model of dental fear, threat attributions patients make during dental visits activate cognitive schemas—deeply ingrained mental frameworks developed from prior experiences and information—centered on uncontrollability, unpredictability, danger, and disgust [24]. These schemas, which increase patients’ sense of vulnerability, fundamentally shape how patients experience dental visits, making the situation seem threatening. In other words, fearful patients often interpret some ambiguous aspects of operatories or encounters as threatening. These threat perceptions activate long-held protection-oriented beliefs (developed from past experiences, stories they’ve heard, or information they’ve processed) that lead them to expect any or all of four main things: that they will not be able to control what happens, that they cannot predict what will occur, that something painful will occur, and that the experience will be disgusting or revolting. These automatic mental patterns create a sense of vulnerability (i.e., the patient feels open to potential harm). Once activated, these thought patterns shape how the patient experiences the entire visit, making even routine procedures feel scary and disorganizing.

#### Expanded Cognitive Vulnerability Model

The four-factor cognitive vulnerability model was derived from evolutionary-based explanations for stimuli (e.g., heights, snakes, enclosed spaces) that generate phobic responses in some individuals [25]. Although this model comprehensively explains non-human threats and certain human-related threats (i.e., unpredictability and uncontrollability), it does not fully capture threats involving people with a duty to ensure safety. From an evolutionary perspective, children’s survival and healthy development depend on having adult protectors who can be relied on to prevent harm [26]. The fundamental need for trustworthy caregivers extends beyond the parent–child relationship to include other caregivers—such as teachers and healthcare providers—who assume an ethical duty to do no harm, actively prevent harm, and work in the best interest of the vulnerable person [27]. When this primal expectation of trustworthiness is violated, it can engender fear and anxiety responses equivalent in intensity to the model’s other four domains [26]. Therefore, the addition of “untrustworthy” as a fifth factor reflects the essential human need for reliable protection from those in positions of authority and care. This factor has repeatedly been cited in approaches viewing dental fear through a psychosocial lens [28–30].

### 1.2. Model Interplay

**Figure 1.**
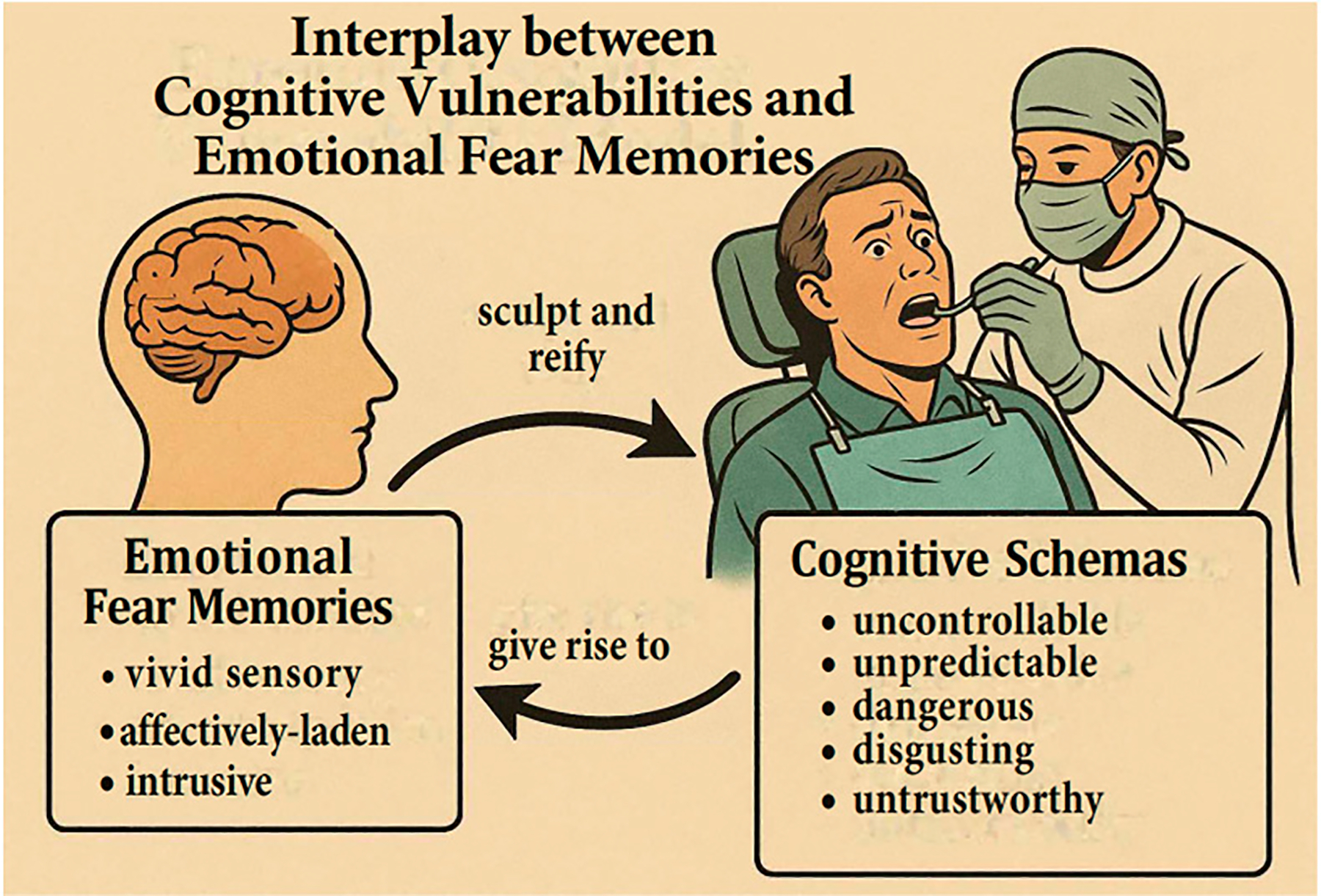
Dental fear etiology model interplay. Represents the interaction between emotional fear memories and cognitive vulnerability schemas in the development and maintenance of dental fear.

Rather than competing explanations of dental fear acquisition, it is likely that the etiology of dental fear implicates both cognitive vulnerabilities and emotional fear memory development for some individuals. Specifically, threat-responsive cognitive processes co-occur with and give rise to the attentional and neurobiological arousal responses [31] that facilitate the consolidation of emotional fear memories. Emotional fear memories, in turn, can sculpt and reify cognitive schemas of dental visits as threatening. See Figure 1.

### 1.3. Current Studies

#### 1.3.1. Study 1

The existing research makes a compelling case for emotional fear memory as a component of dental fear yet leaves significant room for replication and expansion. Most research examining whether negative memories of dental visits have the characteristics of emotional fear memories (e.g., vividness, sensory qualities, affective content) has relied on participants rating these predefined memory qualities on trauma scales. We sought to assess the presence of these characteristics in the content of existing memory narratives shared by individuals who reported experiencing or witnessing a childhood traumatic dental event.

We hypothesized that parents’ deliberately recalled memories of self-described childhood dental traumas would align with the characteristics of emotional fear memories (i.e., include visceral details and sensory descriptors, be affectively laden, and reflect intrusiveness in long-term impacts). Without a priori hypotheses, we also sought to examine (a) whether these reflections included the child’s reactions directed toward the parent or dentist, including blame attributions, and (b) whether these incidents shared common characteristic features.

#### 1.3.2. Study 2

Given the literature suggesting that adult dental fear is often rooted in aversive childhood or adolescent experiences [24], we sought to (a) describe adults’ recall for salient dental events generally and (b) inquire if self-reported worst dental experiences would cluster around childhood or any other specific stage of life. We hypothesized that the quality of memories would vary as a function of self-reported dental fear, with more fearful adults more likely to report their earliest and worst dental visits with the qualities of emotional fear memories (i.e., sensory, affectively laden, intrusive). Because the cognitive vulnerability model of dental fear [32] has received empirical support [4,25,33,34], we also hypothesized that the memories of fearful individuals would be more likely to reflect the vulnerability characteristics of experiencing the dental visit as uncontrollable, unpredictable, dangerous, or disgusting.

## 2. Materials and Methods

### 2.1. Study 1

Study 1 used archival data (collected between January 2004 and September 2007), collected anonymously with verbal consent and no identifiers, descriptors, or demographic information at a private practice specialty clinic. The study team had no interaction with the participants and no access to any private health information (PHI) or other information regarding participants including demographics; thus, the New York University Langone institutional review board ruled that—pursuant to the U.S. Department of Health and Human Services Common Rule [35] (45 CFR § 46.104[d][4])—study 1 was exempt from human subjects review.

#### 2.1.1. Participants and Procedures

Parents (*N* = 17) of dentally anxious children contributed data. Parents were told about the initiative after arriving for their children’s dental appointments. Parents received a sheet of paper with a blank space for them to write; the instructions were as follows: “We are compiling descriptions of traumatic incidents that have occurred involving children in dental offices. The material will be used as a learning tool for young dentists in training, including in presentations or papers about dentistry. No names or identifying information will be used. If you, or one of your children, experienced such an incident and you would like to contribute, please take a few minutes and describe what happened, the long-term effects (if any), and any other details that are important to you.” Individuals who chose to write something left the anonymous forms in a box at the clinic.

#### 2.1.2. Qualitative Analyses

The anonymous, handwritten reflections were transcribed and qualitatively coded by two independent reviewers. Coding was performed based on an a priori coding framework [36] using seven categories: five from trauma literature on emotional fear memories [9,10], in addition to any reference to the developmental period during which the incident occurred, and any reactions directed at the parent or dentist. These categories are demonstrated by the column headings in Table 1. Discrepancies were resolved via consensus during team meetings.

### 2.2. Study 2

This study, approved by the New York University Langone Institutional Review Board (# i23–01494), was conducted between January and March 2025 to further examine the role of memory in dental fear.

#### 2.2.1. Participants and Procedures

Participants (*N* = 58) were recruited in the waiting area of NYU College of Dentistry clinics between January and March 2025. Participants were informed that the study team was interested in assessing what people remember about their dental visits, including childhood visits and unpleasant visits, and whether they experienced current dental fear. Interested patients were privately screened to determine eligibility (i.e., at least 18 years old, able to read and write, had ever visited the dentist) and provided consent for participation. They then completed a paper survey on a clipboard in a corner of the waiting room, handing the paper back to the study facilitator upon completion. Three patients discontinued their surveys prematurely, as they were called for dental appointments. Completion time took less than 10 min for all participants. No names were collected, and participants did not provide any identifying information. Compensation was not provided for this study.

#### 2.2.2. Measures

Dental fear was rated on a modified Gatchel single-item rating scale [37] (“On a scale of 0–10, how do you feel about going to the dentist, where 0 is no fear, 4 is moderate fear, and 10 is extreme fear”), which has been shown to have predictive validity comparable to that of more complex questionnaires [38]. The Gatchel scale has established convergent [39–41], discriminant [39], concurrent [42], and criterion validity [43]; it is not associated with biased socially desirable responding [39]. Single dental fear scales have moderate-to-strong convergence with longer scales in adults [44].

Participants were then asked to “include any details you remember and your age at the time” for two qualitative prompts: “describe your earliest memory at the dentist” and “describe your worst memory at the dentist.”

#### 2.2.3. Qualitative Analyses

Participant surveys were scanned, transcribed, and qualitatively coded based on codes derived a priori from two frameworks [36]. The coding system comprised three codes from study 1 assessing emotional trauma memory qualities and five codes reflecting cognitive vulnerabilities [32] (e.g., dental visits as uncontrollable, dangerous) across both earliest and worst memories. Ages at each experience were also recorded. All responses were independently coded by two trained raters. Raters were trained in behavioral coding by the first and second authors, including didactic instruction in underlying theories, coding practice, and feedback sessions. Percent agreement ranged from 80% to 100%, and discrepancies and final decisions were resolved via consensus during full team meetings. The final codes are shown in Table 2.

## 3. Results

### 3.1. Results—Study 1

#### 3.1.1. Age at Which the Visit Occurred

Most parents (*n* = 13) of dentally anxious children reflected on traumatic dental visits from their childhoods, with a minority reporting on their children’s traumatic dental experiences (*n* = 4). Among participants reporting on their own traumatic childhood experiences, most (70%) included an approximate age during which the event occurred, all of which were reported to happen during early or middle childhood (*M* = 5.11 years old; *SD* = 1.69; range: 3–8 years old). Three of the four individuals reporting on their children’s traumatic dental experiences included their children’s ages, with all three visits occurring during toddlerhood (1–2 years old).

#### 3.1.2. Characteristics of Self-Reported Traumatic Childhood Dental Memories

Every childhood dental memory reported by participants (*n* = 13) included visceral details and sensory descriptors of the dental setting (e.g., “office was sterile and the machines were big and frightening”), instruments and procedure (e.g., “used the little pokey tool on me and then put his finger in my mouth”), the dentist (e.g., “struggling so hard to yank the tooth out [that] he was sweating,” “held down by a big arm over my face”), the patient themselves (e.g., “feeling sick to my stomach”), or other visual (e.g., “vomit in the white porcelain bowl with water swirling around it;” “the root was twice as along at the tooth itself”) or sensory descriptions (e.g., “horrible smell,” “teeth were loose and I could wiggle them,” “my body remembers”). Every memory included explicit affective language reflecting the patient’s fear (i.e., used the words “fear,” “scared,” “afraid,” “terrified,” or “traumatized”), and five additionally included references to pain. Nine participants remembered crying during the procedures, and three remembered feeling nauseated or vomiting.

Despite some references to positive experiences and better care during adulthood (*n* = 4), all participants described long-term effects related to the remembered visit. Two directly referenced continued intrusive qualities (“The experience with the dentist who slapped me stays with me til this day. When I go to the dentist, that memory always comes back”; “still feel like a child in that chair, afraid to say how I’m feeling, even in pain”). Others (*n* = 4) described ongoing physiological manifestations of their dental fear as adults (e.g., ”white knuckled and breathe shallowly,” “break into a cold sweat,” “have a pit in my stomach,” and inability to relax). Behavioral avoidance (“thirty years later and I cancel 3–4× [before] every appointment”) and its repercussions (“To this day, I put off seeing a dentist, with the results being a root canal and broken teeth”) was evident in two reflections, and others indicated an ongoing reliance on anxiety medication to make it through appointments (*n* = 4), continued apprehension despite improvement in fear (*n* = 3), and significant current dental anxiety (*n* = 4). Several narratives included mentions of wanting to break the intergenerational transmission of fear by seeking out better dental experiences for their children (*n* = 4).

#### 3.1.3. The Roles of Dentists and Parents in Participants’ Traumatic Childhood Dental Memories

Three of the narratives referenced deliberate separation from a parent, exacerbating participants’ childhood fears. Across narratives, the dentists’ behaviors are overt contributors to the fear participants experienced as children. Participants described having their faces, mouths, and noses covered (*n* = 3), which one participant referred to as “the smothering;” being held down or restrained (*n* = 4); having one’s cheek grabbed and being physically shaken (*n* = 1); and being slapped across the face by the dentist (*n* = 3). Others reported being screamed or yelled at, told to shut up, and being spoken to unkindly (*n* = 4); additionally, two referenced their dentists telling them they needed to “grow up” or “get brave.” Some participants described experiences of invalidation in dentists not believing them when they reported being in pain or physically ill (*n* = 3), and one described themself as feeling “powerless to that man.” Two attributed some degree of blame to parents, with one reporting feeling “betrayed” by their mother’s failure to comfort them, and another asking why their mother let it happen. See Table 1.

#### 3.1.4. Characteristics of Parent Memories Concerning Their Children’s Traumatic Dental Experiences

Parents’ recollections of their children’s traumatic dental experiences (*n* = 4) did not include the sensory details generally characteristic of emotional fear memories. Memories included mention of (a) procedures and (b) both parents’ and children’s behavior during procedures. All four memories emphasized restraint of the children, which tended to be described in depth, with three parents reporting having held down their children and one refusing to do so. Three described their children’s behavior, including crying (*n* = 2), screaming (*n* = 2), wrestling (*n* = 1), kicking (*n* = 1), and hair-pulling (*n* = 1). Consistent with emotional fear memories, all narratives were highly affectively laden when describing both the children’s and parents’ emotional reactions, with all children described as extremely fearful and parents experiencing shock and sadness during the event (e.g., feeling “horror,” “emotional,” “exhausted,” “crying internally,” and “distanced/shutdown”). The mother who reported feeling shutdown described dissociation during the incident (“… as if I was watching the procedure from afar even though I was speaking and participating”). Two parental reflections overtly invoked self-blame and regret, with these parents perceiving themselves as having failed their children (e.g., “the memory of that procedure, where we let our daughter down and could not help her during a very fearful time, will always haunt me, and I definitely would choose a different option if I could go back; “I broke my child’s trust in me to keep her safe, for the first and last time, and I wish that I had never allowed it to happen”). All four documented the ongoing implications of the experience for their children as aligned with emotional fear memories, including nightmares (*n* = 2), current dental anxiety (*n* = 4), treatment refusal (*n* = 2), and behavioral inhibition in toddlerhood (*n* = 1). See Appendix A, Table A1.

### 3.2. Results—Study 2

#### 3.2.1. Participant Demographics

Over half (55.2%) of participants identified as female (*n* = 32), with 43.1% as male (*n* = 25) and 1.7% choosing not to report (*n* = 1). Participants identified their race as White (*n* = 28; 48.3%), Black or African American (including multiracial) (*n* = 19; 32.8%), Asian (including multiracial) (*n* = 5; 8.62%), and Native American or Alaska Native (including multiracial) (*n* = 1; 1.72%); five participants (8.62%) did not report race. Ethnic identification was *n* = 39 (67.2%) not Hispanic or Latino, and *n* = 12 (20.7%) Hispanic or Latino; *n* = 7 (12.07%) did not report ethnicity. Age ranged from 18 to 83 years (*M* = 49.1, *SD* = 16.0), with *n* = 1 (1.7%) not reporting age.

#### 3.2.2. Age During Experiences

Among individuals who reported age at the earliest experience (67%), the mean earliest remembered visit occurred at 11.0 years old (*SD* = 7.9); age ranged from 3.5 to 49 years; 13 participants failed to include an age in their writeup, and 6 used non-numerical descriptors of a corresponding developmental period (e.g., “child” or “teen”). Time from the earliest remembered dental visit to study participation varied from 1 year (reported by a 50-year-old participant who denied prior visit memories) to 77 years (reported by an 83-year-old participant who recalled a visit at the age of 5); *M* = 35.5 years since the earliest remembered visit (*SD* = 17.9).

The mean age for the worst recalled dental visit (57% reporting) was 29.21 years old (*SD* = 19.33) with a range of 5.5 to 79 years; 19 participants failed to report age at their worst dental visit, and 6 used non-numerical descriptors of a corresponding developmental period. Time from the worst remembered dental experience to study participation ranged from 1 week (reported by a 62-year-old participant) to 70 years (reported by a 75-year-old participant); *M* = 22.7 years since the worst visit (*SD* = 21.5).

#### 3.2.3. Reported Dental Fear

All but one (*n* = 54) of the participants who provided complete memory data rated their current dental fear. Reported dental fear scores ranged from 0 to 10 (*M* = 3.68, *SD* = 2.75), with 26 participants reporting none-to-mild (0–3) fear (46%), 21 reporting moderate (4–6) fear (43%), and 7 reporting severe (7–10) fear (11%).

#### 3.2.4. Worst and Earliest Memory Overlap

Four participants (<1%) reported that their earliest dental memory was also their worst visit. Two of the four endorsed none-to-mild fear, one reported moderate fear, and one reported severe dental fear.

#### 3.2.5. Earliest Dental Visit Memories

##### Positive and Neutral Recollections

Twenty percent (*n* = 11) of participants described their first dental visits or some component of them in overtly positive terms—commenting on the entire visit (e.g., “loved going, “a very pleasant memory”), kid-friendly features of the office (e.g., “highlight was playing on the toy tables;” “a dental hygiene TV show featuring gorillas” that was “very cute”); their dentist (e.g., “I loved that dentist,” “dentist was very funny”), or a reward received (e.g., “they gave me a treat after so it went nice[ly],” “gave us candy,” “gave a toy”). Nine of these participants were in the non-to-mild dental fear category. Only 2 of the 21 participants endorsing moderate dental fear recalled positives of their earliest visits; none of the participants endorsing severe dental fear (n = 7) reported any positives associated with their earliest visits.

An additional 27% of participants (*n* = 15) described neutral experiences but emphasized the lack of negatives associated with the first remembered dental visit. Participants reported few remembered details (e.g., “really can’t recall,” “don’t remember much”); the absence of significant pain, procedures, or apprehension (e.g., “remember it not hurting,” “do not remember much pain,” “not at all scary,” “nothing bad”); or the visit being unremarkable (e.g., “they found nothing,” “uneventful,” “ordinary”). Twelve of these participants endorsed either no current fear or mild dental fear; three endorsed moderate dental fear.

##### Characteristics of Emotional Fear Memories

*Sensory descriptors and visceral details*—positive, negative, and neutral—were evident in 27% of participants’ earliest memories (*n* =15). Memories included auditory (e.g., “sounds of the drill; “sounds of the machines”), gustatory (e.g., “choosing between bubble gum + banana flavor foam at the end, and I picked banana and I liked it”), visual (e.g., “patients spitting into bins with saliva and blood,”), olfactory (e.g., “the strong smell of chemicals;” “the smell of the drill and teeth, and blood”), and tactile (e.g., “anytime I felt pain & would squeeze [mom’s hand],” “how sharp some of the tips [of the tools] were”) descriptors. Other sensory memories were corporeal (e.g., “I realized I’d rather feel the sensation of the drill over the sight of the needle;” “I vomited on his hand, arm, apron”). Five of these descriptors were recalled by participants currently endorsing no dental fear or mild dental fear. Of them, two described the flavors of the toothpaste, one the “pink tablet that showed where I didn’t brush enough,” and another the injury prompting the visit, “faceplant on ice.” Only one included an overtly negative descriptor related to the dental visit (e.g., “It was a government hospital. I heard screaming [… and] I was so afraid and nervous”). Of the ten sensory memories recalled by individuals with current moderate-to-severe dental fear, two were neutral (e.g., “Him making fake teeth … smoothing them, shaping them… Rather than fear, it was more of a curiosity;” “Dentist was very pleasant and he used something … felt like a buzzing sensation, and all my baby teeth fell out”). The remainder were associated with negative experiences (e.g., “hammered on the metal braces… lots of long needles and extractions … [for] teeth on top of teeth and many painful cavities”).

###### Affectively laden memories.

Forty-four percent (*n* = 24) described affectively laden memories. Experiences of recalled pain (*n* = 14) were the most frequent (e.g., “It was painful. I was screaming and begging for help;” “He wouldn’t give me local anesthesia… He drilled out my cavities with an old, belt-drive drill, which was horrible”). Only one participant who recalled being in pain during this early visit reported no current dental fear; indeed, this person wrote about an oral surgery that was “very painful but necessary.” The other thirteen endorsed moderate-to-severe dental fear. Other affective experiences described across memories included fear (*n* = 7; “afraid of the needle and didn’t want it;” “It was very scary”), discomfort (*n* = 2; “Having to open my mouth up very wide was always uncomfortable for me”), and shame (*n* = 1; “I felt ashamed of not being good at caring for my teeth”). Nine of the ten individuals reporting these affectively laden memories endorsed current moderate-to-severe dental fear.

###### Intrusiveness.

A fraction of participants (9%) described memories with an intrusive quality (*n* = 5). They either mentioned reexperiencing (e.g., “My teeth do feel sore now whenever the machines start to work;” “had nightmares;” “Most [dental] experiences since, I’ve felt ashamed”) or avoidance (e.g., “did not see another dentist until I had to, around 40 years old… no actual memories of the visit, but sense the big gaps were due to some unconscious negativity about my dental work … unconscious anxiety;” “I never wanted to go to the dentist after that and cried to my teacher so she could help me not go.”) All five participants reporting memories with intrusive qualities endorsed moderate-to-severe dental fear (*M* = 7.2, *SD* = 2.58).

#### 3.2.6. Worst Dental Visit Memories

##### Positive and Neutral Recollections

When asked to describe memories of their worst dental visits, 7% (*n* = 4) denied ever having negative visits (e.g., “I didn’t have any bad memories … I didn’t have any problems;” “I cannot say I have had bad memories with [the] dentist. I am blessed with good dental health”). All these individuals reported none-to-mild dental fear.

##### Characteristics of Emotional Fear Memories

*Sensory descriptors and visceral details* were evident in 33% of participants’ worst recalled dental visits (*n* = 18). Unlike the early visit memories, all inclusions of sensory details across fear groups were overtly negative for participants’ worst memories. Six of these memories came from participants reporting no or mild dental fear (23% of this group). These included auditory memories (*n* = 3) (e.g., “They turned on a machine that was very loud and made the headache worse”) and unpleasant sensations related to the procedures (*n* = 3) (e.g., “hate[d] the feeling of the water jet hitting soft tissues in mouth”). Twelve of these sensory memories came from participants endorsing moderate-to-severe dental fear (43% of this group). They included brief mentions of sensations associated with an unpleasant visit (*n* = 6) (e.g., “the crunching noise and pulling” (extraction); “the scraping of gums;” “dental guard … makes me want to jump out of my skin”) and entire memories described in sensory and visceral detail (n = 6) (e.g., “Doctor yanked on and cracked tooth. [He] broke it and had to go back in with what felt like plyers. After 1.5 h of complete pain, the tooth was out… I had bones from my jaw coming out weeks later;” “I could still feel the unbearable, vague pain even after anesthesia, and remember the dentist was complaining how deep the roots were. He was sweating, and a long sigh came out of his mouth after it was finished”).

###### Affectively Laden Memories.

Affectively laden worst dental memories were common, with 55% of the sample reporting them (*n* = 31). The majority (*n* = 23) described being in pain or feeling uncomfortable during the procedure (e.g., “Periodontist … did a very painful job, so bad I couldn’t brush my teeth much less floss for a couple of years”). Other emotions experienced included fear and anxiety (“anxious of anesthesia & if surgery would change facial structure; “scared of the big numbing needle”), and shame over having to undergo a root canal (*n* = 1). Of all affectively laden memories, 11 were attributable to participants currently endorsing no fear to mild dental fear (42% of this group); 20 were attributable to participants endorsing moderate-to-severe dental fear (71% of this group).

###### Intrusiveness.

Indicators of intrusiveness were only overt in the recalled worst memories of 5% of participants (*n* = 3). One reported intrusive re-experiencing (e.g., “discomfort of anesthetic needle is a vague memory that covers over a cluster of memories”) and two others reported avoidance following this experience (e.g., “It was years before I returned to the dentist”). All three were participants currently endorsing no fear or mild fear.

#### 3.2.7. Cognitive Vulnerabilities

##### Earliest Memory—Cognitive Vulnerability Themes

Cognitive vulnerabilities were evident in 27% of the earliest recalled dental visits (*n* = 15). Only two of the five possible (uncontrollable, unpredictable, dangerous, disgusting, untrustworthy) vulnerabilities were reflected in these memories. Specifically, the earliest recalled memories included the perception of the dental visit as “dangerous” (*n* = 13) and of the dentist as untrustworthy (*n* = 4).

Among memories with cognitive vulnerabilities represented, three came from participants reporting none-to-mild dental fear (11% of this group). Two of them described “dangerous” visits (i.e., “dentist has broken my front crown” and “a very painful” surgery), and one described her childhood dentist as untrustworthy (i.e., “As an adult, I believe this dentist told my siblings and me that we had cavities to make money”). Eight of the memories reflecting these cognitive vulnerabilities came from participants reporting moderate dental fear (38% of the moderate fear group). Of them, six included “dangerous” perceptions (e.g., “I was 8 years old when I broke my front tooth and had to get a root canal. It hurt a lot and was very scary;” “the dentist did not give me any numbing meds. He just drilled until he reached the desired hole”). One included a perception of the dentist as untrustworthy (i.e., the orthodontist would retaliate by not rinsing his hands of [soap] … they were soapy when he put them in my mouth), and one reflected both perceived dangerousness and untrustworthiness (i.e., “I was 6 years old. That dentist should have been a butcher. [He] created fear and pain. Parents could afford him with payment plans”). Finally, three memories that included dangerous perceptions (“painful without pain relief injections, the older technology machines, the rude dentist—it was painful…a disaster”) and one that included a perception of untrustworthiness (e.g., “He was … a little bit of a jerk….. We had to do x-rays and he was trying to put the bitewing in. I kept saying I would throw up. He didn’t listen and then I vomited on [him]”) came from participants with severe dental fear (71% of this group).

##### Worst Memory—Cognitive Vulnerability Themes

Across all reported worst memories, 58% recalled memories that included cognitive vulnerabilities; the most common was “dangerous” (*n* = 23). Others were “untrustworthy” (*n* = 7) and “uncontrollable” (*n* = 2). Among participants with mild fear, the most common cognitive theme for their worst memory was “dangerous” (*n* = 10). Examples of dangerous cognitions in this group include a “horrible dentist” performing a root canal, “painful visits” where they tightened metal bands in their mouth, and a participant being “scared” about the needle and the unfamiliarity of the space. Other participants described “pain from drill,” an “aggressive dentist,” an “intensely painful” procedure, or a painful root canal where the participant “had to go back about three times. I remember not wanting to go back the second and third times.” The second most common for the mild fear group were “uncontrollable” (*n* = 1) and “untrustworthy” (*n* = 3). The example of an “uncontrollable” cognition in this group was a participant who stated, “The dentist would give me too much gas, and I had no knowledge of what was going on.” Regarding “untrustworthy,” one participant in this group stated, “They tried to sell us on an over $30k treatment plan. It put me off wanting to go to the dentist for a long time.” Another participant recalled that the dentist “pushed a lot of procedures,” and another participant described an event where “The dentist said to lift my hand (and wave) if I felt pain, and he would stop. I did, and he did not stop.”

Among participants with moderate fear, the most common cognitive vulnerability was a perception of visits as “dangerous” (*n* = 12). Examples in this group included being “scared of the big numbing needle,” painful procedures such as “gum surgery,” and a procedure during which the dentist “did a root canal on the wrong tooth. It hurt forever.” Another participant described a scary experience, during which the dentist sexually harassed them: “He was hitting on me with the door closed and then started to take his shirt off when I screamed ‘Stop!’” Other reported themes for the moderate group included “untrustworthy” (*n* = 4) and “uncontrollable” (*n* = 1). Examples of “untrustworthy” cognitions within this group included, “I was told everything was clear from the doctor, when in reality an infection was building up.” Another participant stated, “I was told to put my hand up if there was any pain. But when I did, he told me there was no pain I was feeling.” Another participant “[I was] losing teeth … seeing a dentist who I believe didn’t want to let go of his patients, so when he should have been treating [me] more in depth, he has been re-gluing [the artificial teeth]…disasters over and over.” Regarding the “uncontrollable” theme, one participant described their worst memory as “not being in control of my own mouth.”

Among participants with severe dental fear, one participant reported a “dangerous” cognition, while four did not report any cognitive vulnerabilities in describing their worst memory. In this group, an example of a dangerous cognition is demonstrated by a participant who stated, “The dentist used to be like a terrorist with those older machines [with a] lack of kindness and rude behavior… I kept feeling I am not surviving—they will kill me.” (Some participants with no/mild fear [*n* = 11] and severe fear [*n* = 4] did not report a cognitive vulnerability for their worst memory.) See Table 2.

### 3.3. Collated Results: Fear-Evoking Dental Procedures

The specific dental procedures associated with negative dental memories (for study 2, “worst” visits) were mentioned in narratives by most participants (76%) across both studies. See Table 3. Fillings, followed by extractions, were the most common procedure associated with childhood traumatic memories (study 1). Notably, across procedures, childhood restraint was mentioned in 47% of traumatic visit memories. Root canals, followed by wisdom teeth extractions, were the procedures most commonly reported during worst dental visits (study 2). For individuals currently endorsing none-to-mild dental fear, having never identified any bad visits (12%) was as prevalent as the procedure associated with the worst visits (root canals). For individuals currently endorsing moderate-to-severe fear, wisdom teeth extractions were the procedure most associated with worst visits (18%), followed by root canals and gum procedures (14% each). Across both studies, the most cited adverse visit procedures were root canals (13%), fillings (11%), and extractions (9%).

## 4. Discussion

This two-study paper provides an in-depth examination of dental memories among patients across the spectrum of fear. Individuals’ memories for dental visits were evaluated from two coding frameworks developed a priori: one based on the emotional fear memory literature (studies 1 and 2) and the other inspired by an expanded cognitive vulnerability model of dental fear (study 2). The frameworks are compatible, at times overlapping (e.g., affectively laden pain memories inherently derive from dangerous situations), means for evaluating the qualities of fear-based memories.

Replicating the only other thematic analysis of qualitative dental fear recollections [14], we found that (a) sensory imagery was not uncommon among participants across a range of dental fear (from none to severe) and (b) the content of the imagery (pleasant to overtly negative) tended to vary as a function of fear [14]. Specifically, no study 2 patients endorsing severe dental fear described any pleasant aspects or remarked on the absence of negatives associated with their earliest visit. Furthermore, consistent with our hypotheses, negative sensory details, affect, and intrusiveness in first-visit descriptions were overrepresented among individuals with moderate-to-severe current dental fear. Notably, when asked to describe their worst dental visit (study 2), negative sensory details, affect, and intrusiveness were apparent across fear classifications, but contrary to hypotheses, no differences were evident as a function of fear. This is consistent with prior findings that bad dental experiences are not uncommon and not uniquely predictive of future dental fear [25].

Memories for trauma-endorsing individuals (study 1), rather than for those merely describing worst experiences, were the most vivid and starkly negative of all recollections. Consistent with the trauma literature and our hypotheses, individuals who self-selected into this group universally reported emotional fear memories, characterized by (a) sights, sounds, and physical sensations, (b) negative affect, and (c) intrusiveness (including long-term implications). Strikingly, these adults’ memories of childhood visits (appointments that should be routine, unremarkable, and mostly forgotten 30 years later) are rife with detail. Several of these narratives fit psychiatry’s former criterion of “traumatic events”: threat evoking fear, helplessness, and horror [45]. Indeed, for this group, the implications of these early childhood visits (e.g., impairing fear, constant discomfort, and routine avoidance) have been lifelong.

Informed by research findings that most adults with dental fear experience childhood onset, and our knowledge of stress and memory processes, we wondered whether the worst dental memories would also be the earliest for fearful participants (study 2). However, only four participants in this study reported that their worst memory was also their earliest, and these individuals displayed a range of fear scores, suggesting no clear pattern or relationship. One possible explanation for this lack of association is that our sample was drawn from the general population rather than specifically targeting individuals with severe dental fear, or individuals who reported having experienced a trauma (as in study 1). Given that fearful individuals (who identified their worst experiences as having occurred across ages) did not describe any positive characteristics of early visits, a cognitive bias (that individuals likely to develop fear are also likely to fail to attend to and encode any positives, or that positives are discounted and forgotten over time), as associated with a threat schema, could be at play.

Consistent with limited prior research, references to pain and dentists’ behaviors [24] are prominent foci across trauma memories (study 1) and worst dental memories (study 2). The study 1 narratives (from 2004 to 2007) came from parents recollecting their own childhood experiences, inevitably highlighting some now-disfavored practices (e.g., “hand over mouth exercise” [46]; telling children to grow up and be brave). Although misguided, these practices were more accepted—even recommended—decades ago. Yet other behaviors, such as slapping children across the face as retribution for noncompliance and separating very young children from parents, are difficult to fathom as sincere, well-intentioned behavioral strategies for sound oral healthcare provision. Notably, the few study 1 memories that focused on parents’ experiences all included restraint as a primary theme. Only one other study [47] has considered parents’ opinions in the debate about pediatric dental restraint. Like our findings, a subset of parents in that study expressed feelings of regret and guilt for allowing this, leading the authors to question the ethics of restraint and urge its use only during “extraordinary circumstances” [47]. Our (albeit few) parent recollections, as well as the mention of restraint in participants’ own childhood traumatic memories, echo that recommendation.

Throughout study 2, negative dentist behaviors are largely captured by characterizations of dangerousness and untrustworthiness (the two most represented cognitive vulnerabilities of early and worst memories). Findings regarding cognitive vulnerability themes mirror those for emotional fear memory qualities. For the earliest recalled memories, these characteristics are overrepresented among individuals with moderate-to-severe dental fear (as opposed to the none-to-mild fear group). However, for *worst* memory recall, cognitive vulnerabilities were common across fear categories. Fearful patients’ visit memories seem more likely to routinely share these characteristics. However, a single visit attribution of dangerousness or untrustworthiness of the dentist (along with recalled negative sensory details, affect, and intrusiveness) is not in and of itself sufficient for developing dental fear. A host of other identified risk factors (e.g., developmental stage and visit timing, temperament, biological–genetic factors, prior painful dental experiences, trauma history, and health literacy) [48–50] interact with these processes to predict fear. Additionally, this study corroborates other findings regarding feared procedures. Specifically, root canals and extractions (including wisdom tooth extractions) have been found to elicit high levels of dread, anxiety, and pain in adult patients [51,52], and caries are significant predictors of childhood dental fear [53].

The original cognitive vulnerability model [32] is based on an evolutionary theory of fear acquisition. According to this theory, perceptions of something as uncontrollable, unpredictable, dangerous, and disgusting facilitate the development of the cognitive schema underlying fear [32]. Although this framework has received some support across studies explaining dental fear [32], we found that it was unable to capture a prevailing element of individuals’ fear narratives. That is, an evolutionary-based model of fear, which adequately explains fears of snakes and heights, can be somewhat lacking when the object of fear is interpersonal or interpersonally mediated. Indeed, across earlier studies, we (like other researchers [29,30]) have found support for a prominent social aspect of dental fear that is not fully encompassed in the prospect of ceding control or the potential dangers inherent in the dental visit. The Seattle Model of dental anxiety devoted a specific fear classification (subtype IV) to distrust of dentists [28]. Other research has focused broadly on the socioemotional aspects of dental fear and how lack of trust in the provider can evoke experiences of powerlessness, shame, and embarrassment—all of which exacerbate dental fear severity and avoidant behavior [29]. Similarly, lower scores on the Dental Trust Scale (DTS), the only validated assessment of patients’ trust of dentists, have been associated with current pain, dissatisfaction with care, having changed providers, and past experiences that included fainting, gagging, discomfort, and embarrassment [54]. Therefore, we added the perception of the dentist as “untrustworthy” to the vulnerability model.

Indeed, a substantial portion of our participants described experiences that reflected distrust of the dentist or dental staff (e.g., instances of not being believed when articulating pain, dentists failing to stop a procedure in response to an agreed-upon safety cue, making statements that patients experienced as belittling or humiliating). Most significantly, in some cases, distrust arose from experiences of financial exploitation, during which patients felt that dentists were recommending unnecessary procedures, leading them to question whether the dentist truly had their best interests at heart. This violates the “non-maleficence” principle within healthcare ethics [55]. Yet, it is not unheard of; dental scholars have recognized the increased costs of dental education and financial pressures to adopt cuttingedge technology for patients’ improved comfort and experience as potential contributors to dentists straying from ethics and evidence-based practices [56,57].

For child and adult patients across both studies, details about the most negative visits largely converged on experiences of threat, both in terms of the pain endured and the dentists who would elicit (and often could not be trusted to mitigate) that pain. The salience of these memories (particularly the ones from childhood) to adult patients and the ways their lifelong oral healthcare has been compromised are important lessons for practicing dental providers. Many of these memories also highlight the important role of dentist–patient communication. Participants’ narratives described feeling blindsided by the actual procedures that occurred or ignored by the dental staff when articulating pain or concern. Although they also appeared in adult narratives, they were particularly prominent in childhood memories. Indeed, there is a tendency among some pediatric dental providers to direct conversation at the consenting parent or guardian, at the expense of the child’s understanding [58]. Yet, research demonstrates that children as young as four express a desire to be involved in discussions about treatment, and it is a provider’s responsibility to ensure developmentally appropriate conversations with both parties to prevent fear [58].

This study also extends nascent research examining memory in dental fear, particularly the role of emotional fear memories in anxiety development. It further extends research on Armfield’s cognitive vulnerability model [32]. Prior research used questionnaires that specifically assessed the presence or absence of the four components: uncontrollable, unpredictable, dangerous, disgusting) [25,34,59,60]. Instead, we used qualitative methods to thematically code participants’ open-ended survey responses about dental memories using Armfield’s categories (and the additional category “untrustworthy”). This approach yielded rich qualitative data that enable a better understanding of memories and perceptions of dental appointments and dental staff behavior.

Our study has several limitations. First, although open-ended survey questions allowed participants to share dental memories in their own words, analyzing the spontaneous occurrence of information consistent with specific themes, the format is also limited. As we did not conduct interviews, we were unable to probe more deeply into narratives by asking follow-up questions and were unable to achieve data saturation. Although the qualities of emotional fear memories were naturally present across individuals’ recollections of traumatic experiences for study 1 (as expected), we may have missed capturing greater detail about study 2 participants’ emotional states (and any potential cognitive vulnerabilities) with specific questions. As it was, emotional responses were only captured when participants chose to volunteer that information, potentially leading to underreporting of cognitive vulnerabilities. Second, scientific inquiries of memories necessarily involve retrospective recall biases. Participants’ recollections are not videos; they are encoded through threat-responsive neurobiological processes that prioritize survival-relevant information. Although a limitation of this study is that we cannot establish the veracity of patients’ recall, it is the subjective experience of these memories—not their historical accuracy—that influences current dental fear and avoidance. Finally, cognitive vulnerabilities are not isolated components but are interrelated perceptions that tend to overlap. For example, an experience that feels primarily uncontrollable can also be perceived as unpredictable and dangerous due to the uncontrollability; an experience that involves trust violations (e.g., refusing to honor stop signals) can also imply uncontrollability. In our analysis, however, we focused on identifying the most explicit and prominent cognitive vulnerability present in each memory for simplicity and consistency across the data.

Findings allude to the profound impact of early childhood dental memories, particularly those associated with fear and trauma, on lifelong oral healthcare. To further validate and expand these findings, several complementary directions for future research are recommended. One important direction is to move beyond qualitative insights into the cognitive vulnerabilities of “uncontrollable,” “unpredictable,” “dangerous,” “disgusting,” and “untrustworthy” experiences by developing and utilizing standardized quantitative measures for these perceptions. This would allow for more precise measurement of their prevalence across different populations, enabling researchers to track how these vulnerabilities are elicited by specific dental experiences, thereby offering a clearer picture of their role in the development and maintenance of dental fear. Quantifying these vulnerabilities would also be crucial for systematically evaluating the effectiveness of interventions designed to address them, such as enhanced communication, fostering patient control, or actively building trust.

Prospective longitudinal studies that begin in early childhood would allow researchers to track the formation and evolution of dental memories, examining how emotional fear memory characteristics develop and persist. Given that our research highlights the enduring nature of early traumatic dental memories and that most adults with dental fear experienced its onset in childhood, such studies could pinpoint specific early dental experiences or memory characteristics that predict later dental fear.

Mixed-methods studies that combine qualitative memory narratives with validated quantitative measures of the cognitive vulnerabilities are also warranted. This approach would allow for a more systematic examination of relationships between memory characteristics and fear severity, while providing rich qualitative data for understanding perceptions of dental appointments and dental staff behavior. Finally, intervention studies could test whether memory-focused therapeutic approaches, such as memory reconsolidation techniques or narrative therapy, more effectively reduce dental fear when combined with traditional exposure-based treatments.

## 5. Conclusions

Given that the weighted prevalence of dental fear across the seven U.S. studies conducted in clinical practices is 38.87% [61] and that memories of traumatic dental care are remarkably vivid and tend to have lifelong implications, we make the following recommendations based on our study’s results. First, dental practices should universally screen every patient at every visit for dental fear on a simple 0–10 scale. Second, for fearful patients (4+ on the 0–10 scale) [37], dental professionals should inquire about prior negative experiences at the dentist, acknowledging and validating the patient’s experiences. Third, the dental staff should partner with the patient to plan an approach that minimizes situations evoking the primary vulnerabilities underlying the patient’s prior experiences (i.e., staff should listen for themes of uncontrollable, unpredictable, dangerous, disgusting, and untrustworthy events and actions). Finally, dental healthcare providers should diligently enact the plan, providing an opportunity to disconfirm cognitions [62] that these themes are endemic and unavoidable when seeking dental care.

## Figures and Tables

**Table 1.** Parents’ recollections of traumatic dental visits they experienced as children.

Age	Incident Details	Affect Named	Patient Reaction	Reactions Directed at Parent/Dentist	Intrusiveness and Implications
4	“**Office was sterile and machines were big and frightening.**” “He attempted to examine my teeth and I began to cry.” “Mother, rather than comforting me, became angry.”	scared, terrified, betrayed	cried; became hysterical	“I’m sure she was embarrassed but it left me feeling betrayed and terrified.”	“To this day, I put off seeing a dentist, with the results being a root canal and broken teeth. I still feel like a child in that chair, afraid to say how I’m feeling even in pain.”
nr	“**My body remembers**” “She was not allowed to accompany me…; forced to be separated from my mother was such a traumatic experience at such a young age.” “Dentist telling my mother, ‘She’ll have to grow up sometime.’”	shy, scared	“I cried terribly. I cried and cried and ended up vomiting on dentist.”		“I go every 6 months for cleanings but am often **white knuckled and breathe shallowly**…never would I call it a pleasant experience.”
6	“I was very scared and uncooperative. I was held down and yelled at … **A hand was held over my nose and mouth and nose**. … I can still **remember dentist yelling ‘get the gas,’ which made me more scared because I knew my grandparents’ relatives were gassed** in Poland in WWII.”	scared; terror	“I was fighting so hard and so in terror. I managed to run out of the office.”		“To this day I’m not a fan of going to the dentist.”
6	“Family dentist decided to pull several teeth that were not coming out…**I remember some of my teeth were loose and I could wiggle them.** Some were definitely not loose” “I was first afraid of the needle used to inject the Novocain, then **I felt the pain** and the doctor didn’t believe me. He kept telling me to stay still and asked for assistance from his staff to keep me in the chair” “Get brave, the dentist kept saying.”	afraid; pain	“I cried and was hysterical.”	“Two things were clear. It hurt and he didn’t believe me.” “I have since learned I metabolize Novocain rapidly. It was not ‘in my head’ as dentist kept saying to me”	“I have difficulty relaxing even for cleanings.” “I have improved over the years” (but described extensive coping procedures [breathing, meditations] and meds to get through). “I believe none of these special needs would be necessary if I had a better start with my first experiences at the dentist.”
nr	“Trip to the dentist with a big smile on your face and leaving in fear and tears asking why.” “I was sitting thinking what will happen, and **what happened next stayed in my memory**. The **dentist was screaming at me to sit straight. The next thing I know she had pulled my tooth. It was painful.**”	pain; fear	“I cried for two hours.”	“I kept asking my mom why.”	“Even now I’m trying not to be afraid…I know it’s bad. I still have fear…and with my kids I’m afraid it will be them crying…because I don’t want them to have the same experience as me that they will remember for a long time.”
3	“It was my first checkup He wouldn’t let my mother come back with me even though I was very scared and crying. I had cavities and he started to fill them right there. He gave me a shot of Novocain and I was hysterical. **He couldn’t calm me down while he was drilling so he slapped me across the cheek**. I was totally silent and petrified for the rest of the time.”	very scared; petrified	“Hysterical; stunned silence”	“I didn’t tell my mother for 2 years. I thought that’s just what happens when you go to the dentist.”	“Nervous wreck every time I go to the dentist. Even though it’s 30 years later and I get good dental care as an adult, I have a **pit in my stomach** whenever I’m at the dentist’s office.”
6	“I was getting a filling and made a crying noise. The dentist **grabbed the inside of my cheek, shook my head, and told me to shut up**”	fear	crying		“To this day, I break out in a **cold sweat** before the dentist. I think I passed fear onto my daughter.”
3	“We lived in a small town with only one dentist. My dad had died in May. I got this toothache in October. **Dentist used the little pokey tool on me and then he put his finger in my mouth** to check the tooth and I bit him. He slapped me in the face.”	traumatized	bit dentist		“Dentists used meds to calm or sedate me until I was old enough for nitrous—still use it today.” “The experience with the dentist who slapped me stays with me til this day. When I go to the dentist, that memory always comes back.” “I worry about my children being traumatized and keep moving dentists.”
nr	“I used to **feel sick to my stomach** whenever I had an appointment. I remember them **drilling and just crying** because it **hurt so much** and always being told, ‘It’s almost over, not long now’”	“pain, fear and more fear”	crying; nausea	“As a kid, never believed me when I told them it hurt.”	“At 36 still hard for me to go;” takes 3 x standard numbing agent as an adult.”
6	“I had my first cavity. I was **held down** by a nurse **while I kicked and screamed**. I received no anesthesia.… told to just hold still and be quiet. I **remember the horrible smell and pain to this day**,”	pain, traumatized	kicked, screamed	“I was talked to very meanly by the dentist.”	“I’m over 40, and it has taken me years to heal my dental trauma and to realize I could have a positive dental experience fear.”
4	“I had an abscess on my gum. I still remember the trauma and fear I felt that day at the dentist. **I remember seeing needles on the tray and trying to climb out of the chair**.” “I would vomit every time because of the fluoride. I couldn’t make it out of the office. I’d **vomit in the white porcelain bowl with water swirling around it next to the dentist’s chair**.”	fear	tried to climb out of chair; vomit	“The dentist never believed me when I said I was going to throw up; always did.”	“To this day I’m apprehensive about going to the dentist.”
nr	“My first memory is of having a baby tooth pulled…I don’t remember if I was given anesthetic, **but I remember the dentist struggling so hard to yank the tooth out he was sweating**…I remember **the root of the tooth was twice as long as the tooth itself**,” “The dentist and his assistants would **cover our mouths and noses whenever we were scared and crying**. Once my sister needed a filling. When the smothering didn’t calm her, the dentist asked for permission to put my sister in a straight-jacket.”	terrified, scared	crying		“I’ve been terrified at the dentist for as long as I can remember despite having nice thoughtful dentists for the past 20 years. Thirty years later and I cancel 3–4 x every appointment. I’m still so nervous I take Valium and have nitrous and anesthesia.”
8	“Having to get a cavity filled. I remember feeling scared and **being held down by a big arm over my face**. I felt overwhelmed by the drilling and began to cry. I wanted my mom. I was told no. When I protested and cried louder, I was slapped which scared me into submission.”	scared; overwhelmed	crying	“I felt powerless to this person. I told my mom and we never went back. I’m not sure what she did or said to them, but I was glad we never had to return.”	“I’ve had years of dental work and never had an experience that bad again. Now that I have a daughter, I want to make sure she has only positive ones.”

Note: descriptive sensory imagery and visceral details in **bold**.

**Table 2.** Cognitive vulnerability model [25] themes (plus “untrustworthy”) from worst and earliest dental memories.

Dental Fear Rating (Modified Gatchel (0–10)	Age at Earliest Recalled Dental Visit	Age at Worst Recalled Dental Visit	Are Earliest and Worst Recalled Memories the Same?	Earliest Memory Cognitive Vulnerability Themes	Worst Memory Cognitive Vulnerability Themes
0	10	38	No	Untrustworthy	Uncontrollable
0	8	12	No	N/A	N/A
0	“Teenager”	N/A	No	N/A	Dangerous
0	9	9	Yes	N/A	N/A
0	N/A	N/A	No	N/A	N/A
0	49	49	Yes	Dangerous	Dangerous
0	N/A	N/A	No	N/A	N/A
0	15	69	No	Dangerous	N/A
0	10	N/A	No	N/A	N/A
0	“Kid, young”	“Teen”	No	N/A	Dangerous
0	7	12	No	N/A	Dangerous
1	5.5	22	No	N/A	Dangerous
1	5	N/A	No	N/A	N/A
1	5.5	“Teen”	No	N/A	Untrustworthy
2	18	N/A	No	N/A	N/A
2	5.5	16	No	N/A	N/A
2	N/A	22	No	N/A	Dangerous
2	N/A	18	No	N/A	Dangerous
2	N/A	25	No	N/A	Dangerous
2	N/A	“Adult”	No	N/A	Dangerous, Untrustworthy
2	3.5	5.5	No	N/A	Untrustworthy, Dangerous
3	N/A	N/A	No	N/A	N/A
3	8	N/A	No	N/A	N/A
3	N/A	N/A	No	N/A	N/A
3	19	N/A	No	N/A	N/A
4	6–10	26	No	N/A	Dangerous
4	“Child”	“Last few years”	No	N/A	Untrustworthy
4	5–6	N/A	No	Dangerous	N/A
5	13	N/A	No	N/A	Uncontrollable, Dangerous
5	N/A	N/A	No	N/A	Dangerous
5	16.5	N/A	No	N/A	Untrustworthy
5	16	22	No	N/A	N/A
5	13	N/A	No	Dangerous	Untrustworthy
5	7.5	22	No	N/A	Dangerous
5	6	28	No	N/A	N/A
5	7	N/A	No	Dangerous	N/A
5	15	25	No	Untrustworthy	Dangerous
5	10	65	No	N/A	Dangerous
5	10	“Adult”	No	Dangerous	Dangerous
5	N/A	79	No	N/A	N/A
5	N/A	14	No	Dangerous	N/A
5	18	45	No	N/A	Untrustworthy
6	6	22	No	Dangerous; Untrustworthy	Dangerous
6	“Pediatric”	35	No	N/A	N/A
6	Childhood	12.5	No	N/A	Dangerous
6	8	8	Yes	Dangerous	Dangerous
6	9	22	No	N/A	Dangerous
7	8.5	38	No	Untrustworthy	Dangerous
7	7.5	N/A	No	N/A	N/A
8	N/A	N/A	N/A	N/A	N/A
8	“Child”	62	No	Dangerous	N/A
8	17.5	“recently”	No	N/A	N/A
10	N/A	N/A	Yes	Dangerous	Dangerous
10 *	8	24	No	Dangerous	N/A

Note: cognitive vulnerability themes: uncontrollable, unpredictable, dangerous, disgusting, untrustworthy;

* participant indicated that “until [New York University (NYU) clinic] it was 10. At NYU clinic, it is 4”.

**Table 3.** Dental procedures associated with traumatic and most negative memories.

Dental Procedure	*Study 1*		*Study 2*		Across Studies
	Traumatic Experiences	None-to-Mild Fear	Worst Experiences Moderate-to-Severe Fear	Study 2 Total	
unspecified	2	9	6	15	**17**
root canal	2	3	4	7	**9**
filling	6	1	1	2	**8**
extraction	4	1	2	3	**7**
wisdom teeth extraction		1	5	6	**6**
exam	3	2	1	3	**6**
gum procedures (surgery, grafting, I&D)			4	4	**4**
not applicable		3		3	**3**
cleaning		2	1	3	**3**
orthodontic tightening		2	1	3	**3**
repair of restorations (crowns, veneers)		1	2	3	**3**
restoration			1	1	**1**
X-rays		1		1	**1**
Total	17			54	**71**

*Note*: Across procedures, childhood restraint is mentioned in 8 of 17 trauma memories reported for study 1.

## Data Availability

The key qualitative data presented in this paper for study 1 are all included in the presented tables (Tables 1 and A1). Full records for study 2 are available upon request from the corresponding author (after signing a data-use agreement).

## Appendix A

**Table A1.** Parents’ recall of their children’s traumatic visits (N = 4).

Age	Details Excerpted	Affect Named	Child’s Reaction	Parents Experience (Thoughts, Feelings)	Implications
**1**	“my son was into nursing at night and developed bottle teeth … dentist said we needed to do a pulp … took about 45 min and we (my wife and myself) had to hold him down. It was pure horror for all involved.”	“pure horror **for all** involved”	worst experience of son’s life	“I’m a paramedic and not easily put off my medical practices. “one of the worst experiences of my adult life”	“He is quite a shy boy now at age 2. We trace it back to this day…”
**2**	“…I felt rushed and uncomfortable. Daughter was scared.” “He had me hold her down .… opening her mouth and trying to prepare for the numbing injections and daughter was struggling and crying… assistant to hold her head still so he could inject her. I saw her begin to bleed as he injected her several times with the numbing shot “ “back into the chair and being held down by me, she started screaming and crying. I was holding her arms down, and my husband held her legs down because she was kicking and struggling violently to get free while she screamed. “ she jumped into my lap still crying and screaming. “That’s why we only do 2 teeth at a time,” said Dr. X.” “looked in prize box…Oh, good,” said Dr. X. “That means she was just scared since she stopped crying so quickly.” He paused for a minute and then said, “She’s actually not the worst one we’ve had here by far.”	**child**: fear; terror; confusion **mom:** shut down and distanced	crying, kicking, screaming; she never stopped crying	“Purposefully keeping her crying and scared “was tearing me up” “I felt shut down and distanced, as if I was watching the procedure from afar, even though I was still speaking and participating.” “I broke my child’s trust in me to keep her safe, for the first and last time, and I wish that I had never allowed it to happen.	“ride back home, daughter seemed very distant from us. It was as if a bond had been broken. Later that night in her sleep, she started crying and screaming. She reattached herself to us but the night terrors kept happening for a few weeks afterwards, then began tapering off. She also became upset every time we wanted to brush her teeth or mentioned the dentist and would say “No dentist” and “No teeth”, cover her mouth and refuse to brush. Some time after this, she had an accident on the playground and we took her to my husband’s dentist to make sure her teeth were okay, and once she saw the dental equipment in the back of the office immediately became terrified and started crying, saying “No Dentist” took her to second appointment and stopped because she was terrified. “She still has night terrors, bolting up in the middle of the night saying “No!” but less often than she used to. I am now convinced that traumatic dental visits have very negative effects.”
**2**	daughter was “extremely apprehensive toward being worked on. My husband and I were asked to hold her down or they would use a papoose to finish. We chose to hold her down.” “husband held her legs.”	**child:** extreme fear; **parents:** exhaustion	screamed, wrestled, pulled mom’s hair	“I cried internally the entire time” “My husband and I were unsure if we had done the correct thing” “the memory of that procedure, where we let our daughter down and could not help her during a very fearful time will always haunt me, and I definitely would choose a different option if I could go back.”	Since her first experience, my daughter has an extreme fear of the dentist and resists all treatment. My husband and I have always felt that her early experience set her up for failure in her future dental visits; years of dental anxiety (now age 9)
**NR**	[daughter’s] “first trip to the dentist to pull a tooth was very emotional for us both. They put a mask on her with laughing gas. She cried and cried and said she couldn’t breathe, so we left” “another dentist … very abrasive and asked me to hold her down. I couldn’t do it, so we left” “once again a horrible experience” “asked me to leave room after I would not hold her down”	**child:** afraid **parent:** emotional	cried and cried	“They were very upset and said I was the problem and that if I would have left the room they would have been able to pull her tooth out. I could never do that”	she had nightmares about it and never wanted to go back; now both of my daughters are afraid of the dentist

## Abbreviation

The following abbreviations are used in this manuscript:

DTS: Dental Trust Scale
